# The Influence of the COVID-19 Pandemic on the Hearing Impaired

**DOI:** 10.7759/cureus.31348

**Published:** 2022-11-10

**Authors:** Marzouqi A Salamah, Asma Al-Ahmadi, Sahal Arabi, Waleed A Alsaleh, Abdullah Aljuwayyan, Medhat F Yousef, Roa Halawani, Abdulrahman Hagr

**Affiliations:** 1 Otolaryngology - Head and Neck Surgery, King Abdullah Ear Specialist Center (KAESC) College of Medicine, King Saud University Medical City (KSUMC) King Saud University, Riyadh, SAU; 2 Otolaryngology - Head and Neck Surgery, Ohud Hospital, Al-Madinah Al-Munawarah, SAU; 3 Audiology, King Abdullah Ear Specialist Center (KAESC) College of Medicine, King Saud University Medical City (KSUMC) King Saud University, Riyadh, SAU; 4 Audiology Unit, ENT Department, Faculty of Medecine, Menoufia University, Shibin Al Kawm, EGY; 5 Otolaryngology - Head and Neck Surgery, Ohud Hospital, Riyadh, SAU; 6 Otology, King Abdullah Ear Specialist Center (KAESC) College of Medicine, King Saud University Medical City (KSUMC) King Saud University, Riyadh, SAU

**Keywords:** face mask, social distancing, hearing loss, hearing aid, covid-19

## Abstract

Background

In this study, we aimed to investigate the effect of the coronavirus disease 2019 (COVID-19) pandemic-induced social restrictions, including face masks, on patients with hearing problems.

Methodology

This cross-sectional survey study was conducted in an ENT tertiary care center. After signing the consent form, we invited study subjects with hearing disabilities who were using unilateral or bilateral hearing aids to participate in filling out the study survey. The study questionnaire was completed by 80 subjects. The questionnaire included various questions about the respondent’s demographics, hearing aids, and communication with a face mask during COVID-19 restrictions. All statistical analyses were performed using SPSS version 19 (IBM Corp., Armonk, NY, USA).

Results

Overall, 40% of the study sample agreed that understanding people wearing face masks is harder because their speech is muffled, whereas 10% disagreed, and 50% were neutral. While 41.3% agreed that understanding is harder because they cannot see their mouth moving, 23.8% disagreed, and 35% were neutral. More than half of the study sample (55%) agreed that they are worried about how they will communicate with others if wearing face masks becomes more common. However, 50% of the participants thought that they can still hear people when they speak to them from a safe distance. Of note, 71.3% of subjects disagreed about tinnitus being worse since the lockdown.

Conclusions

The widespread use of face masks had a significant impact on the daily communication and interactions of people with hearing impairments. More research is needed to find creative ways to help these patients improve their daily communication and social interactions.

## Introduction

Coronavirus disease 2019 (COVID-19) is a devastating infectious disease that originally started in Wuhan and rapidly affected the entire world afterward. COVID-19 has led to drastic changes in people’s lives, work, and physical and mental health [1]. Most countries have adhered to the latest preventive measures to curb the spread of the virus. Some of the measures included using personal protection, such as gloves, social distancing, and face coverings, such as face shields and face masks. Moreover, some countries have made face masks mandatory in public areas to fight against the spread of COVID-19 as much as possible [2], which will likely continue for a long time.

Face coverings in public spaces make speech sound more reduced and somehow difficult as people cannot see the lip movement or facial expressions needed for understanding and communication [3]. While understanding speech when people wear face masks is challenging for everyone, it is even more challenging for people with hearing difficulties to interact and communicate with others [3]. These difficulties can lead to an increased desire for social isolation and depression, especially when considering the safety measures applied by local governments worldwide to combat the COVID-19 pandemic [4,5].

The possible negative effects of face masks on social communication and interactions have been reported in the literature. For instance, Trecca et al. investigated the impact of wearing face masks by medical staff on patients with hearing disabilities attending a hospital visit. Of note, mild-to-severe problems were reported in most subjects (86.4%); the major face mask problems were the inability to read lip movement (n = 33), followed by the attenuated sound due to the mask (n = 26) [6]. In another survey-based report by Naylor et al., the authors reported widespread speech understanding problems when using face masks among people with hearing problems and those using hearing aids. However, there was no link between the severity of hearing loss and the degree of communication difficulties because of the face masks [7].

According to the published literature, we hypothesized that face coverings could affect communication and interaction, especially among people suffering from hearing loss. Thus, we conducted this survey-based study among subjects with hearing problems who wear hearing aids. The survey contained several questions that aimed to elucidate the communication and social interaction problems during the COVID-19 pandemic when people wear face masks.

## Materials and methods

Study subjects

This survey-based, cross-sectional study was conducted in our tertiary hospital after obtaining the Institutional Review Board (IRB) approval (H-03-M-084, IRB 61-2020). We invited a total of 120 subjects with hearing disabilities, for which they were wearing unilateral or bilateral hearing aids, to participate in the study and fill out the study survey. There were no limitations regarding age, gender, ethnicity, or any other specific characteristic. The respondents did not receive any kind of incentive for their participation and completion of the survey.

Data collection and questionnaire

Study subjects were invited online to obtain their consent to participate by filling out the study survey. There were no potential risks associated with this study. The participants were asked to fill out a Google Forms questionnaire, no identifying information was obtained from the participants, and all responses were recorded anonymously. Only the authors of this research had access to the data. The survey contained several questions such as the demographics of the respondent, hearing aids, and communication through a face mask during COVID-19 restrictions. This survey was previously utilized in a similar cross-sectional study in Scotland [7]. The questionnaire was developed in an iterative yet timely manner and has been reviewed by a group of experts. It has been translated and validated to facilitate the filling process for the patients. Participants were asked to subjectively rate their hearing without the use of their hearing aids. They had the following four choices: very poor, poor, moderate, and adequate hearing. Those who reported moderate or adequate hearing were combined under the subgroup “better hearing” while participants who reported poor and very poor hearing without hearing aids were combined under the subgroup “worse hearing.” We considered agree and strongly agree as “agreement,” while disagree and strongly disagree were deemed as “disagreement.” Regarding participants who reported “agree to somehow,” their responses were considered “natural.”

Statistical analysis

All statistical analyses were performed using SPSS version 19 (IBM Corp., Armonk, NY, USA). Descriptive statistics were used to outline the characteristics of respondents using frequencies and percentages for categorical variables. We used the chi-square test to determine the significant difference in variables among the subgroups. P-values below 0.05 were considered statistically significant.

## Results

A total of 80 participants completely responded to our structured questionnaire. The majority of respondents (52.7%) were in the age group of 18 to 29 years old, followed by respondents older than 60 years (16.3%). The male gender predominated in the sample (62.5%). Unilateral hearing aids were used by 43 (53.8%) participants, while bilateral hearing aids were used by 37 (46.3%) participants. Regarding the duration of wearing hearing aids, the majority of the participants used them for more than eight hours per day (66.3%). However, only 13.75% and 12.5% used hearing aids from one to four and four to eight hours per day, respectively (Table 1). Regarding hearing performance, as shown in Table 2, 40% of our sample agreed that understanding people wearing face masks is harder because the speech is muffled, of which almost similar percentages were found in the better and worse hearing subgroups with no significant difference (p = 0.9).

**Table 1:** Basic demographic characteristics of surveyed subjects.

	All (n = 80)	Better hearing (n = 24, 30%)	Worse hearing (n = 56, 70%)
Age (years)			
<18	6 (7.5%)	1 (4.2%)	5 (8.9%)
18–29	42 (52.7%)	12 (50%)	30 (53.6%)
30–39	7 (8.8%)	3 (12.5%)	4 (7.1%)
40–49	6 (7.5%)	1 (4.2%)	5 (8.9%)
50–59	6 (7.5%)	3 (12.5%)	3 (5.4%)
>60	13 (16.3%)	4 (16.7%)	9 (16.1%)
Gender			
Male	50 (62.5%)	17 (70.8%)	33 (58.9%)
Female	30 (37.5%)	7 (29.2%)	23 (41.1%)
Site of hearing aids			
Unilateral	43 (53.8%)	19 (79.2%)	24 (42.9%)
Bilateral	37 (46.3%)	5 (20.8%)	32 (57.1%)
Duration of wearing hearing aids			
One hour per day	5 (6.3%)	2 (8.3%)	4 (7.1%)
One to four hours	11 (13.75%)	8 (33.3%)	3 (5.4%)
Four to eight hours	10 (12.5%)	5 (20.8%)	5 (8.9%)
More than eight hours	53 (66.3%)	9 (37.5%)	44 (78.6%)

**Table 2:** Responses to survey questions.

	Total			Better			Worse			P-value
	Agree	Disagree	Neutral	Agree	Disagree	Neutral	Agree	Disagree	Neutral	
Understanding people wearing face masks is harder because their speech is muffled	32 (40%)	8 (10%)	40 (50%)	10 (41.7%)	2 (8.3%)	12 (50%)	22 (39.3%)	6 (10.7%)	28 (50%)	0.9
Understanding people wearing face masks is harder because I can’t see their mouths moving	33 (41.3%)	19 (23.8%)	28 (35%)	8 (33.3%)	10 (41.7%)	6 (25%)	25 (44.6%)	9 (16.1%)	22 (39.3%)	0.047
I think key workers should be supplied with clear (transparent) face masks	41 (51.2%)	22 (27.5%)	17 (21.3%)	10 (41.7%)	10 (41.7%)	4 (16.7%)	31 (55.4%)	12 (21.4%)	13 (23.2%)	0.178
Wearing a face mask interferes with wearing my hearing aid(s)	24 (30%)	39 (48.8%)	17 (21.3%)	7 (29.2%)	11 (45.8%)	6 (25%)	17 (30.4%)	28 (50%)	11 (19.6%)	0.86
I am worried about how I will communicate with others if wearing face masks become more common	44 (55%)	23 (28.7%)	13 (16.3%)	13 (54.2%)	9 (37.5%)	2 (8.3%)	31 (55.4%)	14 (25%)	11 (19.6%)	0.3
When people speak to me from a safe distance, I can still hear them well enough	40 (50%)	16 (20%)	24 (30%)	14 (58.3%)	1 (4.2%)	9 (37.5%)	26 (46.4%)	15 (26.8%)	15 (26.8%)	0.067
It is a relief not to be obliged to attend social gatherings where I won’t hear well	54 (67.5%)	19 (23.8%)	7 (8.8%)	13 (54.2%)	9 (37.5%)	2 (8.3%)	41 (73.2%)	10 (17.9%)	5 (8.9%)	0.162
The possibility of having to speak to people wearing face masks or from a distance adds to my anxieties about going to public places (e.g., parks and supermarkets)	34 (42.5%)	37 (46.3%)	9 (11.3%)	9 (44.6%)	15 (62.5%)	0	25 (44.6%)	22 (39.3%)	9 (16.1%)	0.049
I use video calls (Facebook, FaceTime, Google, Skype, Zoom, etc.) more often now than I did before the lockdown began	23 (28.7%)	30 (37.5%)	27 (33.8%)	11 (45.8%)	7 (29.2%)	6 (25%)	12 (21.4%)	23 (41.1%)	21 (37.5%)	0.087
I am more worried than usual about what to do if my hearing aids stop working, or if I can’t get batteries	52 (65%)	14 (17.5%)	14 (17.5%)	13 (54.2%)	2 (8.3%)	9 (37.5%)	39 (69.6%)	12 (21.4%)	5 (8.9%)	0.006
Since the lockdown began, I have been wearing my hearing aids less than usual	23 (28.7%)	48 (60%)	9 (11.3%)	11 (45.8%)	10 (41.7%)	3 (12.5%)	12 (21.4%)	38 (67.9%)	6 (10.7%)	0.066
I think about my hearing loss more often than usual before the pandemic	13 (16.3%)	53 (66.3%)	14 (17.5%)	5 (20.8%)	16 (66.7%)	3 (12.5%)	8 (14.3%)	37 (66.1%)	11 (19.6%)	0.627
Televised updates about COVID-19 are easy for me to follow	46 (57.5%)	16 (20%)	18 (22.5%)	11 (45.8%)	8 (33.3%)	5 (20.8%)	35 (62.5%)	8 (14.3%)	13 (23.2%)	0.142
Radio updates about COVID-19 are easy for me to follow	39 (48%)	23 (28.7%)	18 (22.5%)	10 (41.7%)	10 (41.7%)	4 (16.7%)	29 (51.8%)	13 (23.2%)	14 (25%)	0.239
My tinnitus has been worse since the lockdown started	14 (17.5%)	57 (71.3%)	9 (11.3%)	6 (25%)	14 (58.3%)	4 (16.7%)	8 (14.3%)	43 (76.8%)	5 (8.9%)	0.247

Further, 41.3% (n = 33) of the sample agreed that understanding people wearing face masks is harder because they cannot see their mouth moving; eight participants were in the better hearing subgroup (33.3%), and 25 participants were in the worse hearing subgroup (44.6%), with a marginally significant difference (p = 0.047). More than half of the responders (n = 41) agreed that healthcare workers should be supplied with clear (transparent) face masks; most of them were participants with worse hearing (n = 31), with no significant difference between both subgroups (p = 0.178). However, 48.8% (n = 39) of our sample disagreed that wearing a face mask makes it hard to use hearing aids. Of these, 28 were in the worst hearing subgroup, and 11 were in the better hearing subgroup, with no significant difference between the two groups (p = 0.86).

Regarding behavior concerns, more than half of the sample (55%) agreed that they were worried about how they will communicate with others if wearing face masks becomes more common; 55.4% and 54.2% of the worse and better hearing subgroups, respectively, contributed to this agreement. Moreover, no significant difference was noted between the two groups (p = 0.3). Nevertheless, 50% of participants thought they can still hear people when they speak to them from a safe distance, and no significant difference was found between the better and worse hearing subgroups (p = 0.067). A total of 54 (67.5%) participants reported their agreement about not being obliged to attend social gatherings where they will not hear well; more than two-thirds of participants in the worse hearing group contributed to this agreement (n = 41, 73.2%) compared with half of the participants with better hearing (n = 13, 54.2%), with no significant difference between both groups (p = 0.162). Moreover, with an approximate similar percentage of both total agreement (n = 34, 42.5%) and disagreement (n = 37, 46.3%), participants reported their anxieties about going to public places with the possibility of having to speak to people wearing face masks or from a distance. However, the majority of participants with better hearing disagreed (n = 15, 62.5%) compared with the majority of participants with worse hearing who agreed (n = 25, 44.6%), and a marginally significant difference was noted between groups (p = 0.049). On the other hand, a total of 11 (45.8%) participants with better hearing reported that they use video calls more often now than before the lockdown began, while a total of 23 (41.1%) participants with worse hearing reported their disagreement. However, no significant difference was noted (p = 0.08).

**Figure 1:**
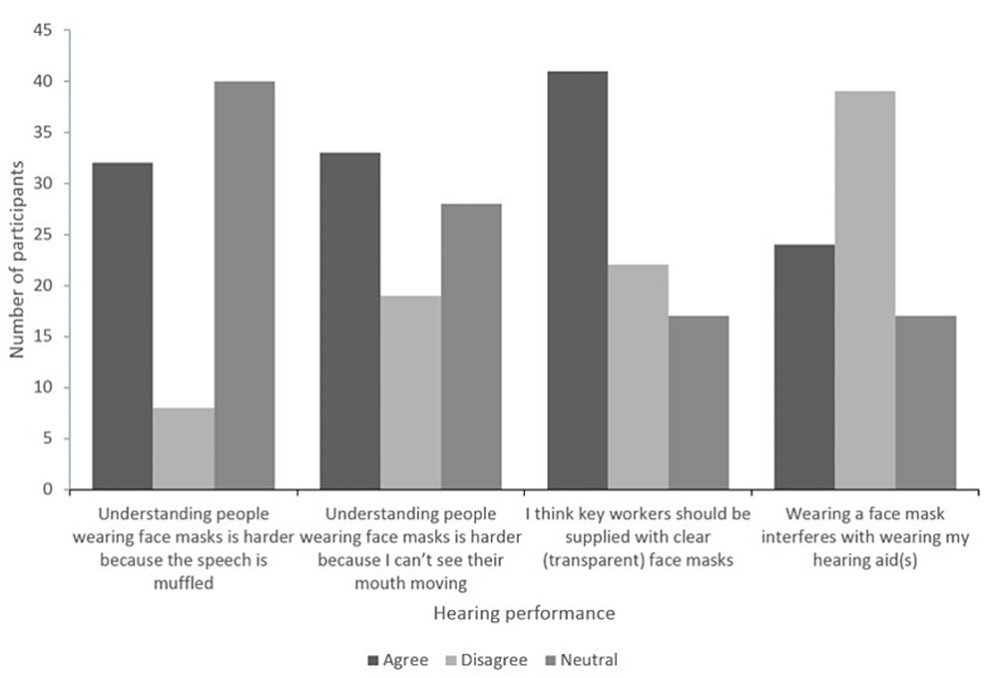
Subjects’ responses regarding hearing performance when people wear face masks.

**Figure 2:**
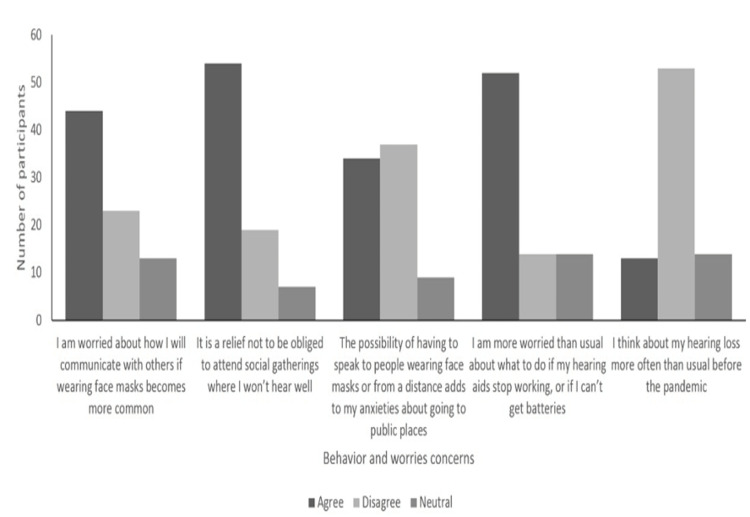
Behaviors and worries due to the widespread use of face masks.

Regarding participants’ increased worries about hearing aids stopping working or not being able to get batteries, 52 (65%) participants agreed on this concern; of those, 69.6% (n = 39) of the participants with worse hearing compared with 54.2% (n = 13) in the better hearing group, with a significant difference (p = 0.006). Moreover, 60% (p = 0.066) and 66.3% (p = 0.627) of the participants reported their disagreement about wearing hearing aids less than usual before the lockdown or thinking about hearing loss more often than usual before the pandemic, respectively, with no significant difference between both groups (Figures 1, 2). Of note, almost half of the sample reported that televised (57.5%) and radio (48%) updates about COVID-19 are easy to follow, with no significant difference between both groups (p = 0.142 and 0.239, respectively).

Regarding tinnitus, more than two-thirds of the sample (71.3%) disagreed about tinnitus being worse since the lockdown began, with no significant difference (p = 0.247) between both groups (Table 2).

## Discussion

The spread of the COVID-19 pandemic worldwide has urged local public health authorities to mandate the use of face masks to curb the transmission of the virus. The widespread use of face coverings has impacted communication, especially among people with hearing problems. The findings of this survey-based report elucidate the effect of face covering and COVID-19 restrictions on people’s communication and behavior. Using face masks impacts daily communication in several ways due to the diminished acoustic power of sound and the lack of lip movement and facial expressions, as has been reported in numerous studies [8-10]. According to a recent study, face masks allow low-frequency sounds to pass while attenuating high-frequency sounds [2]. Face masks decrease the sound quality of a speaker, make speech perception more intricate, make facial expressions inaccessible, and prevent the visual cues necessary for lipreading, which are essential elements of good communication [9]. Several studies have illustrated the role of visual features in speech understanding [8,9,11]. For example, one study found that 70% of communication is done through lipreading and facial expressions [11]. This could be more challenging and a major problem for people with severe-to-profound hearing loss as they depend on lipreading and facial expressions to communicate [12]. In our study, approximately 41.3% of the participants agreed that understanding people wearing face masks is harder because they cannot see their mouths moving, and more than half of the responders agreed that healthcare workers should be supplied with clear (transparent) face masks. A study by Atcherson et al. compared standard masks with transparent masks to check the impact on speech understanding in noise among subjects with or without hearing problems. Of note, there was no difference between both masks among people with normal hearing; however, those with hearing loss reported better performance when using clear masks [13]. In another two studies, the patients attributed the communication problems with surgical masks to their inability to read lip movement [6]. This means that providing access to reading lips with clear masks may enhance speechreading, improve communication, and counteract the attenuated sound [14]. However, transparent masks are not problem-free. It has been said that they are harder to hear out of, steam up, and cannot be washed like cloth masks [15]. In our study, a considerable number of participants reported feeling anxious about communication using face masks and feeling relieved when not obliged to attend a meeting with face masks. In a previous study that examined the impact of face masks on daily life communication among patients with a cochlear implant during the COVID-19 pandemic, the study participants tended to feel more lonely when face masks were used in public spaces [3]. Feeling lonely due to face masks could add to the fact that there has been an increase in loneliness because of the security measures applied by local governments [4,5]. In our study, the majority of subjects disagreed that tinnitus had become worse during the COVID-19 pandemic. In a previous report by Naylor et al., the authors reported that about 42% of the subjects in the worse hearing group agreed that tinnitus had worsened since the lockdown started [7]. However, the severity of tinnitus was not assessed in their study; thus, no conclusion can be drawn.

Our study has some limitations. First is the small sample of enrolled subjects; the low response rate and small sample size may be attributed to the fact that we used an online survey. It is true that not everyone has access to social media and the internet. This is probably going to comprise some of the most susceptible individuals to communication problems because of face masks. Further research should investigate if this group of patients has special needs that have not been reported in our survey. Another limitation is that our survey did not include questions regarding different types of face masks or coverings. While the use of face masks is becoming more prevalent across Saudi Arabia, it is possible that several types of face coverings will emerge. Thus, it is recommended that future studies consider the impact of different types of face masks on communication and interactions among people with hearing problems. Upcoming studies are recommended to further check if the widespread use of face masks impacts communication or decreases anxiety and embarrassment. Another factor to be considered in future research is looking for additional solutions to enhance patient communication, such as transparent face coverings, face shields, and other novel solutions.

## Conclusions

The COVID-19 pandemic has led to a lot of problems for people with hearing problems. Face masking and social distancing affect how people communicate, which is already considered difficult among these patients. Face masks not only impact communication among people with hearing loss but may also increase their worries, anxiety, and loneliness. The results of our survey represent a call to policymakers and specialized manufacturers to produce communication-friendly face masks for healthcare workers to enhance their communication and address their patients’ needs. With the problems caused by the COVID-19 pandemic, more research is needed to find ways to help people with hearing problems communicate better and have a better quality of life.
